# MeHg Developing Exposure Causes DNA Double-Strand Breaks and Elicits Cell Cycle Arrest in Spinal Cord Cells

**DOI:** 10.1155/2015/532691

**Published:** 2015-12-17

**Authors:** Fabiana F. Ferreira, Dib Ammar, Gilian F. Bourckhardt, Karoline Kobus-Bianchini, Yara M. R. Müller, Evelise M. Nazari

**Affiliations:** ^1^Departamento de Biologia Celular, Embriologia e Genética, Centro de Ciências Biológicas, UFSC, Campus Universitário, Trindade, 88040-900 Florianópolis, SC, Brazil; ^2^Instituto de Ciências Naturais Humanas e Sociais, UFMT, Avenida Alexandre Ferronato 1200, Setor Industrial, 78557287 Sinop, MT, Brazil; ^3^Centro Universitário Católica de Santa Catarina, Rua Visconde de Taunay 427, Centro, 89203-005 Joinville, SC, Brazil; ^4^Departamento de Fisioterapia, Centro de Ciências da Saúde e do Esporte, UDESC, Rua Pascoal Simone 358, Coqueiros, 88080-350 Florianópolis, SC, Brazil

## Abstract

The neurotoxicity caused by methylmercury (MeHg) is well documented; however, the developmental neurotoxicity in spinal cord is still not fully understood. Here we investigated whether MeHg affects the spinal cord layers development. Chicken embryos at E3 were treated* in ovo* with 0.1 *μ*g MeHg/50 *μ*L saline solution and analyzed at E10. Thus, we performed immunostaining using anti-*γ*-H2A.X to recognize DNA double-strand breaks and antiphosphohistone H3, anti-p21, and anti-cyclin E to identify cells in proliferation and cell cycle proteins. Also, to identify neuronal cells, we used anti-NeuN and anti-*β*III-tubulin antibodies. After the MeHg treatment, we observed the increase on *γ*-H2A.X in response to DNA damage. MeHg caused a decrease in the proliferating cells and in the thickness of spinal cord layers. Moreover, we verified that MeHg induced an increase in the number of p21-positive cells but did not change the cyclin E-positive cells. A significantly high number of TUNEL-positive cells indicating DNA fragmentation were observed in MeHg-treated embryos. Regarding the neuronal differentiation, MeHg induced a decrease in NeuN expression and did not change the expression of *β*III-tubulin. These results showed that* in ovo* MeHg exposure alters spinal cord development by disturbing the cell proliferation and death, also interfering in early neuronal differentiation.

## 1. Introduction

Mercury is a metal of known toxic properties which occurs naturally and anthropogenically in the environment [1, 2]. Methylmercury (MeHg) is an organic chemical form of mercury that has been widely studied due to its neurotoxic effects in humans and animal models, especially when the exposure occurs prenatally [3–6].

MeHg is able to cross the blood-brain barrier, which is immature in embryos and fetuses, leading to an increased vulnerability of the fetal brain to the toxic effects of this organic mercury [7–9]. Moreover, MeHg tends to accumulate in fetuses due to their inability to excrete this metal [10].

Development of the central nervous system (CNS) is slow and gradual and involves the differentiation and migration of neuronal and glial cells to organize cellular layers that comprise the brain and spinal cord. Thus, damage to the developing CNS caused by MeHg can result in irreversible morphological and physiological impairments, which compromise postnatal motor coordination, learning, and memory functions [3, 11–14].

The effects of MeHg exposure on the CNS are related, particularly in adults, to neurochemical changes that include disturbances in the calcium and glutamate homeostasis [15, 16], reactive oxygen species generation, and oxidative stress in the brain of mammals [17–19]. Additionally, the mitochondrial dysfunction accompanied by the expression of apoptotic proteins has also been identified as an effect of MeHg-induced neurotoxicity [20–22].

Although neurotoxicity caused by MeHg is well documented, developmental neurotoxicity is still not fully understood. A previous work has shown that* in ovo* MeHg exposure results in behavioral impairments, such as anomalous movements and low exploratory activity, as well as morphological and biochemical changes, including alterations in the organization of the cerebellar cortical layers and the increase of the antioxidant enzyme activity in the cerebellum of MeHg-exposed chicks [23]. In this study, we offer a new approach to the exploration of developmental neurotoxicity induced by MeHg, using a chicken embryo as a model. The aim of this study was to investigate the impact of MeHg on the cellular layers of the spinal cord, mainly focusing on cell proliferation and cell cycle. The spinal cord was chosen because the cellular organization of its three layers is less complex when compared to the brain and cerebellum, which makes it easier to characterize the CNS cellular dynamics and thus developmental neurotoxicity. Moreover, there is a lack of knowledge about the developmental neurotoxicity induced by MeHg in spinal cord because the most studied impairments caused by this metal mainly relate to the brain and cerebellum.

## 2. Materials and Methods

### 2.1. Eggs and Embryos

Fertilized eggs of* Gallus domesticus* were obtained from a commercial hatchery (Tyson Foods Brasil Ltda., Brazil). The eggs were weighed (66.6 ± 4.7 g) and transferred to an incubator at 37.5–38.0°C and 65.0% humidity. Prenatal acute MeHg exposure was performed at embryonic day 3 (E3), that is, 20 HH stage series [24]. The embryos received a single dose of 0.1 *μ*g of methylmercury II chloride (Sigma-Aldrich, USA) diluted in 50 *μ*L of saline solution administered into the yolk sac near the vitellin vessels. Untreated control embryos received only 50 *μ*L of saline solution. The dose of MeHg used in this study was determined according to Heinz et al. [25, 26] and on the basis of a previous study performed by Carvalho et al. [23]. After treatment, each egg was returned to the incubator, and embryos were monitored daily* in ovo* up to embryonic day 10 (E10), that is, 36 HH. At E10, the embryos were anesthetized by cooling to 4°C for 15–20 min, removed from the egg shell, and washed in saline solution. All experiments were carried out according to the Ethics Committee for Animal Research of the Universidade Federal de Santa Catarina, Florianópolis, Brazil (Approval number 355/CEUA/UFSC).

### 2.2. Routine Microscopy Techniques and Morphometry of the Spinal Cord

Whole embryos at E10 (*n* = 7 embryos per group of three independent experiments) were fixed in 4% formaldehyde and the trunk region was dissected, embedded in paraffin, and cut into serial transversal sections (6 *μ*m). The sections were stained with hematoxylin-eosin (HE) to analyze the spinal cord morphology and to determine the distribution and morphology of cells in the ependymal, mantle, and marginal layers. The thicknesses of the ependymal, mantle, and marginal layers were measured using a morphometric eyepiece (Olympus, USA) (200x) in midline region of the spinal cord.

### 2.3. Autometallography Method

We evaluated mercury deposition by the autometallography (AMG) method, in which dewaxed sections were immersed in the AMG developer solution (60% gum arabic, 10% potassium citrate buffer, 30% hydroquinone, and 0.5% silver nitrate) in darkness for 60 min. Next, the spinal cord sections (*n* = 7 embryos per group of three independent experiments) were washed with tap water and counterstained with hematoxylin. Mercury deposition was evaluated as brown stained cells, which represent silver surrounding the deposited mercury [27], and classified as absent (−), mild (+), moderate (++), and intense (+++) by an investigator who was blind to the treatment assignments according to Müller et al. [28].

### 2.4. Immunohistochemistry

To evaluate the effect of prenatal acute MeHg exposure, we first looked for proliferation, cell cycle, and DNA damage in the spinal cord using antibodies rabbit anti-phosphohistone H3 IgG (1 : 250; Upstate, USA), rabbit anti-cyclin E IgG (1 : 100, Santa Cruz Biotechnology, USA), mouse anti-p21 IgG (1 : 100, Santa Cruz Biotechnology, USA), and rabbit anti-*γ*-H2A.X IgG (1 : 50, Santa Cruz Biotechnology, USA). Next, we investigated neuronal differentiation using antibody mouse anti-neuronal nuclei NeuN IgG (1 : 100, Chemicon International, USA) and mouse anti-*β*III tubulin IgG (1 : 100, Promega, USA) (*n* = 7 embryos per group of three independent experiments). Endogenous peroxidase activity was stopped with 0.3% hydrogen peroxide in methanol. The sections were washed with 0.1 M phosphate-buffered saline (PBS) pH 7.4 + 0.3% Triton X-100 and then blocked with 5% fetal bovine serum (FBS) in PBS. The sections were incubated overnight at 4°C with primary antibodies, washed with PBS, and then incubated for 90 min at room temperature with peroxidase-conjugated secondary antibody anti-rabbit IgG (1 : 400, Sigma, USA) and biotin-conjugated antibodies, including anti-rabbit IgG (1 : 200, Sigma, USA) and anti-mouse IgG (1 : 200, Sigma, USA). Next, the sections were washed in 0.1 M PBS, and binding sites of antibodies were revealed with 3,3′-diaminobenzidine (DAB) (Sigma-Aldrich, USA). For immunofluorescence, Alexa Fluor 488 goat anti-mouse IgG (1 : 200, Life Technologies, USA) and Alexa Fluor 568 goat anti-rabbit IgG (1 : 100, Life Technologies, USA) were used. Next, the sections were incubated with 4,6-diamidino-2-phenylindole dihydrochloride (DAPI) (Sigma-Aldrich, USA) for 3 min at room temperature. Negative controls of immunohistochemical reaction were treated in the same way, except that the primary antibodies were replaced with 0.1 M PBS.

### 2.5. TUNEL Assay

We used TdT-mediated dUTP nick end labeling (TUNEL) staining to identify apoptotic cells in the dewaxed spinal cord sections (*n* = 7 embryos per group of three independent experiments). TUNEL staining was conducted with a TdT-FragEL DNA Fragmentation Detection Kit (Calbiochem, USA) according to the manufacturer's instructions. TUNEL-stained cells displayed dark-brown precipitates in their nuclei.

### 2.6. Quantitative Analysis

To quantify immunoreactive cells (phosphohistone H3 and NeuN) and TUNEL-positive cells, stereological analysis was performed using the M-42 test system (Weibel number 2, Tonbridge, UK) (200x). The numerical density per area (NA) of the cells was determined according to Mandarim-de-Lacerda [29]. Five random fields of the spinal cord were counted for each section (3 sections per embryo, 7 embryos per group of three independent experiments). In order to quantify the *γ*-H2A.X-positive cells, the integrated density of pixels of the fluorescence digital images was determined using Image J software (NIH Image). A scale bar was determined, and the measurement of the integrated density of pixels was determined in a 3,599.77 *μ*m^2^ frame.

### 2.7. Flow Cytometry

Dissected and unfixed spinal cords were homogenized and submitted to consecutive washes with 0.1 M PBS pH 7.8 (*n* = 7 embryos per group of three independent experiments). Then, the cells were dissociated using 0.25% trypsin for 15 min at 37°C and added to 5% FBS under agitation. Subsequently, the samples were centrifuged at 640 ×g for 10 min and the supernatant was collected [30], incubated with primary antibodies rabbit anti-cyclin E IgG (1 : 1000, Santa Cruz Biotechnology, USA), mouse anti-p21 IgG (1 : 1000, Santa Cruz Biotechnology, USA), and mouse anti-*β*III tubulin IgG (1 : 1000, Promega, USA) for 1 h, and then incubated for 45 min with the secondary antibodies Alexa Fluor 568 anti-rabbit IgG and Alexa Fluor 488 anti-mouse IgG (1 : 1000, Life Technologies, USA). Analyses were separately conducted for each antibody in each treatment. Previously, a run with unstained cells was performed to determine the gates of cells of interest. Additionally, propidium iodide was used to refine the gates of interest. Thus, from the dot plot with 20,000 events and considering the parameters side scatter (SSC-A) and forward scatter (FSC-A), the gates with 1,800 events were determined. FACSCanto II flow cytometer (BD Biosciences, Canada) was used for the analysis. The values are presented in absolute count.

### 2.8. Statistical Analysis

Quantitative data were analyzed using Statistica 10.0 for Windows. The differences between MeHg-treated and untreated control embryos were evaluated by Student's unpaired* t*-test. All data were expressed as mean ± SEM, and *P* < 0.05 was considered statistically significant.

## 3. Results

### 3.1. Mercury Deposition in the Spinal Cord

**Figure 1:**
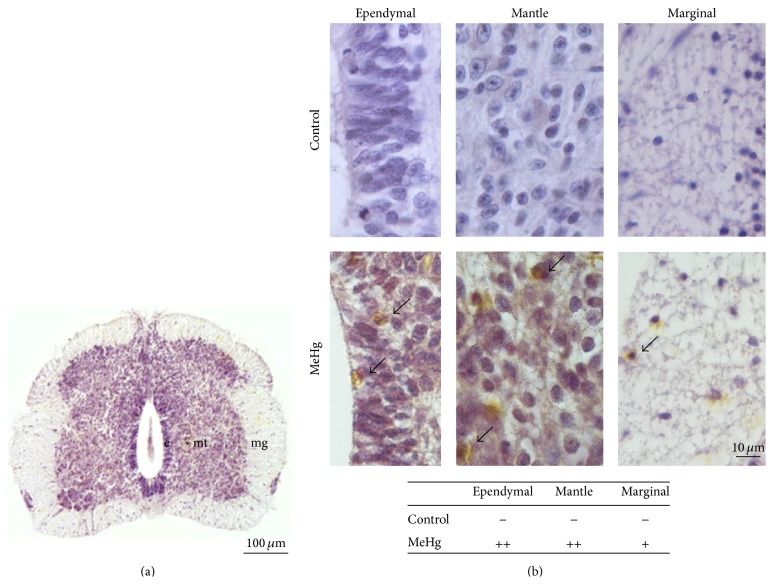
Mercury deposition in the spinal cord of E10 control and MeHg-treated embryos examined by the AMG method. Spinal cord showing ependymal (e), mantle (mt), and marginal (mg) layers in low magnification (a) and high magnification (b). The brown color corresponds to the mercury deposits in cells (arrows) in MeHg-treated embryos. The accompanying table displays the intensity of mercury deposition in the cells of the spinal cord layers, classified as absent (−), mild (+), and moderate (++).

The AMG method was used to show that a single injection of 0.1 *μ*g MeHg into the yolk sac could reach the embryo and its spinal cord. As expected, the mercury deposition was evident in all MeHg-treated embryos but was not observed in the control embryos. The MeHg deposition was recognized in three spinal cord layers, and a greater intensity of deposition was found in the ependymal and mantle layers (Figure 1).

### 3.2. Spinal Cord Morphology and Morphometry

**Figure 2:**
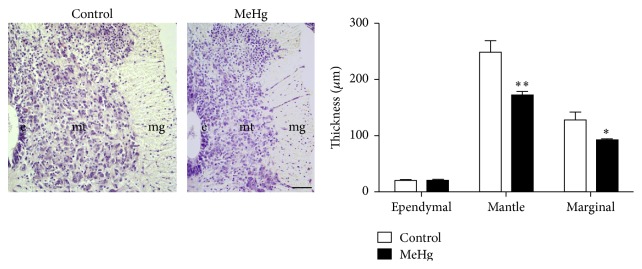
Thickness of the spinal cord layers in embryos at E10. Observe the evident difference in the size of the spinal cord between the control and MeHg-treated embryos. The graph displays the effect of MeHg treatment on the ependymal, mantle, and marginal layers. Data are expressed as mean ± SEM. ^*∗*^ *P* < 0.05 and ^*∗∗*^ *P* < 0.01. e: ependymal layer; mg: marginal layer; and mt: mantle layer. Scale bar: 10 *μ*m.

Considering the fact that the mercury was incorporated in the embryonic tissues, we evaluated the general features of the spinal cord. Similar features of the cell morphology and distribution in the ependymal, mantle, and marginal layers were observed between MeHg-treated and control embryos (Figure 2). However, MeHg treatment caused a significant decrease in the thickness of the mantle layer (172.01 *μ*m ± 7.41) when compared to control embryos (248.19 *μ*m ± 21.48, *P* < 0.01). The same effect was observed in the marginal layer of MeHg-treated embryos (92.03 *μ*m ± 4.12) in comparison to controls (127.74 *μ*m ± 14.74, *P* < 0.05). Only the thickness of the ependymal layer did not change after MeHg treatment between the control (18.94 *μ*m ± 1.14) and MeHg-treated embryos (20.77 *μ*m ± 0.38, *P* > 0.05).

### 3.3. Effect of Mercury on Proliferation and Cell Cycle

**Figure 3:**
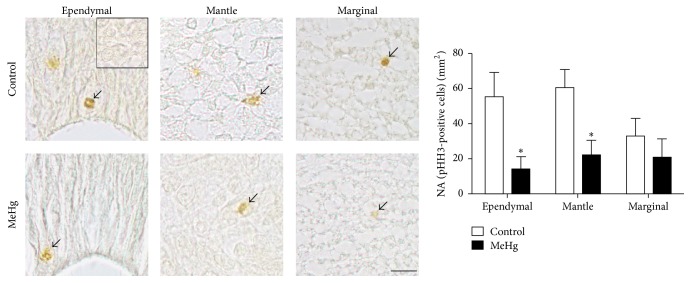
Effects of MeHg on the spinal cord layers of embryos at E10. Proliferating cells labeled with anti-phosphohistone H3 (arrows) were observed in the ependymal, mantle, and marginal layers of the control and MeHg-treated embryos. The square in the ependymal control embryo represents the negative control of immunohistochemical reaction. The accompanying graph displays the NA of proliferating cells, obtained by stereological analysis, in each layer of the spinal cord. Data are presented as mean ± SEM. ^*∗*^ *P* < 0.05. Scale bar: 10 *μ*m.

On the basis of the reduced size of the spinal cord caused by MeHg exposure, the next step was evaluating the proliferation and cell cycle, which are essential for development progress. A few proliferating cells were observed in all embryos, and, after treatment, MeHg caused a decrease in the number of these cells per area. In the ependymal layer, the NA of proliferating cells was significantly lower in MeHg-treated embryos (13.75 cells/mm^2^  ± 7.3) when compared to controls (55.01 cells/mm^2^  ± 13.7, *P* < 0.05). In the mantle layer, the highest number of proliferating cells was observed in the control (60.17 cells/mm^2^  ± 10.4) in comparison to MeHg-treated embryos (21.82 cells/mm^2^  ± 8.6, *P* < 0.05). No significant difference was observed in the marginal layer between the control (32.64 cells/mm^2^  ± 10.2) and MeHg-treated embryos (20.5 cells/mm^2^  ± 10.5) (Figure 3).

**Figure 4:**
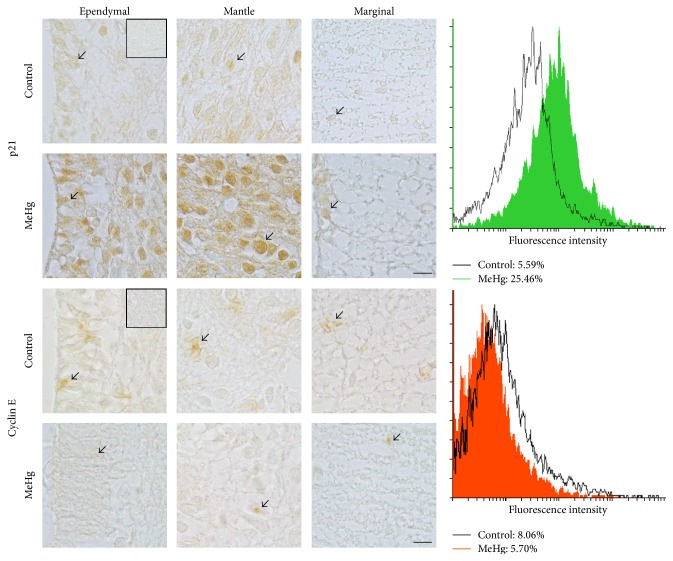
Cell cycle proteins analyzed by immunohistochemistry and flow cytometry. Immunohistochemistry revealed p21 and cyclin E-positive cells (arrows) in the control and MeHg-treated embryos. The square in the ependymal control embryos represents the negative control of immunohistochemical reaction. The graphs display the expression profile and relative frequency of positive cells of p21 and cyclin E in the spinal cord of the control and MeHg-treated embryos. For each treatment 1,800 events were analyzed per antibody. Scale bars: 10 *μ*m.

Moreover, we examined the proteins p21 and cyclin E that play a crucial role in the progression of cell cycle. MeHg caused an increase in the absolute number of p21-positive cells in the spinal cord of treated embryos (473.33 cells ± 62.40) when compared to controls (100.67 cells ± 17.47, *P* < 0.05). However, no changes were observed in the number of cyclin E-positive cells between MeHg-treated (102.7 cells ± 15.2) and control embryos (145.0 cells ± 55.28, *P* > 0.05).  Figure 4 display the immunolocalization and percentage of p21 and cyclin E-positive cells in MeHg-treated and control embryos.

### 3.4. DNA Damage and Apoptosis

**Figure 5:**
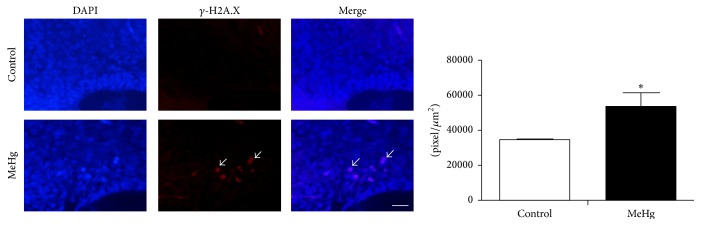
DNA double-strand breaks labeled with *γ*-H2A.X antibody analyzed by immunohistochemistry. *γ*-H2A.X-positive cells (arrows) were observed in MeHg-treated embryos. The graph displays the expression profile of *γ*-H2A.X in the spinal cord of control and MeHg-treated embryos. Integrated density of pixels of the fluorescence of the *γ*-H2A.X was determined, and data are expressed as mean ± SEM. ^*∗*^ *P* < 0.05. Scale bar: 10 *μ*m.

In order to verify if MeHg causes DNA damage, the expression of *γ*-H2A.X protein in response to DNA double-strand breaks was examined. After treatment, MeHg caused an increase in the expression of *γ*-H2A.X (53,740.89 ± 7,834.14 *μ*m^2^) when compared to the control embryos (34,473.89 ± 670.97 *μ*m^2^, *P* < 0.05). These anti-*γ*-H2A.X-positive cells were found mainly in the transition zone between the ependymal and mantle layers of the MeHg-treated embryos (Figure 5).

**Figure 6:**
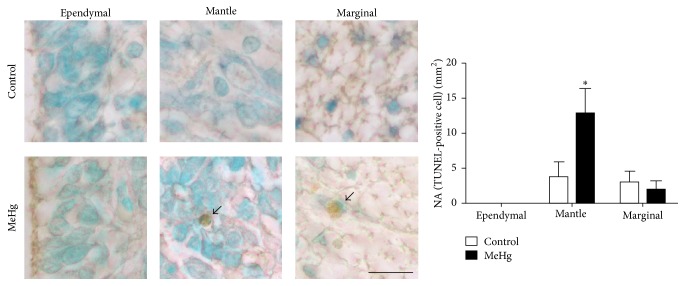
Apoptotic cells in the spinal cord of chicken embryos at E10 recognized by the TUNEL method. MeHg-treated and control embryos showed apoptotic cells (arrows) in the mantle and marginal layers. The accompanying graph displays the NA of TUNEL-positive cells in each spinal cord layer. Data are presented as mean ± SEM. ^*∗*^ *P* < 0.05. Scale bar: 10 *μ*m.

Regarding the increase of DNA damage, we investigated the occurrence of apoptotic cells after MeHg treatment. Few apoptotic cells were observed in the spinal cord of the control and MeHg-treated embryos. A significantly high NA of TUNEL-positive cells was observed in the mantle layer of MeHg-treated embryos (12.73 cells/mm^2^  ± 3.6) when compared to controls (3.82 cells/mm^2^  ± 2.1, *P* < 0.05). However, no significant differences were found between the control (3.09 cells/mm^2^  ± 1.5) and MeHg-treated embryos (2.05 cells/mm^2^  ± 1.2) in the marginal layer. Additionally, no TUNEL-positive cells were observed in the ependymal layer of either the control or MeHg-treated embryos (Figure 6).

### 3.5. Effect of Mercury on Neuronal Differentiation

**Figure 7:**
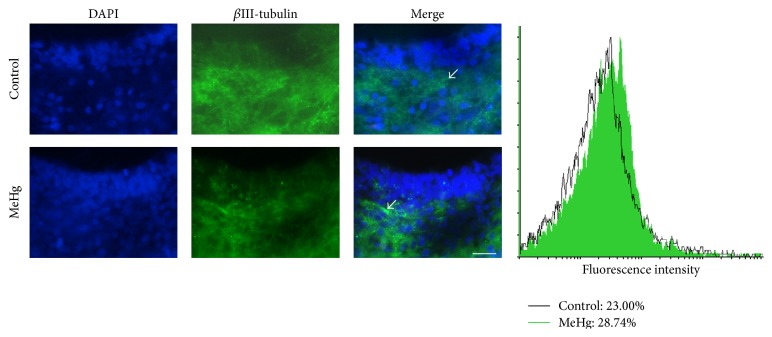
*β*III-tubulin protein analyzed by immunohistochemistry and flow cytometry. *β*III-tubulin-positive cells (white arrows) observed mainly in the mantle layer. The graph displays the expression profile and relative frequency of *β*III-tubulin in the spinal cord of the control and MeHg-treated embryos. Scale bar: 20 *μ*m.

**Figure 8:**
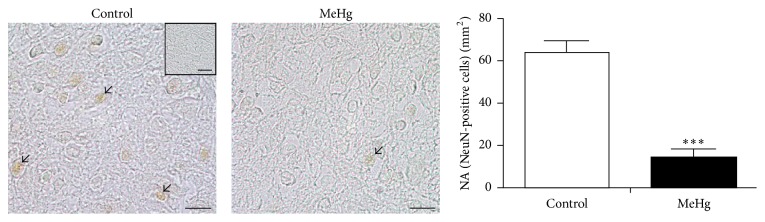
NeuN, a neuron-specific nuclear protein, analyzed by immunohistochemistry. Positive cells (arrows) were found in the mantle layer. The square in the control image represents the negative control of immunohistochemical reaction. The graph displays the NA of NeuN-positive cells. Bars are represented as mean ± SEM. ^*∗∗∗*^ *P* < 0.0001. Scale bars: 10 *μ*m.

We investigated whether MeHg compromises the neuronal differentiation in the developing spinal cord, considering that the major effects of MeHg were observed mainly in the mantle layer, where intense cell differentiation occurs. Immunofluorescence using anti-*β*III-tubulin antibody revealed the expression of this protein in the mantle and marginal layers at E10. After treatment, no changes were observed in the expression of *β*III-tubulin between the control (424.0 cells ± 40.91) and MeHg-treated embryos (517.33 cells ± 1.85, *P* > 0.05) (Figure 7). Additionally, when we analyze the expression of NeuN, recognized in postmitotic neurons and/or during neuronal differentiation, a significantly decrease in the NA of NeuN-positive cells was observed in the mantle layer of MeHg-treated embryos (14.32 cells/mm^2^  ± 4.1) when compared to controls (64.55 cells/mm^2^  ± 5.5, *P* < 0.0001) (Figure 8).

## 4. Discussion

The neurotoxicity of MeHg is a well-known phenomenon, and the present study contributes new data to improve the current understanding of the embryonic cell responses after a single* in ovo* injection of 0.1 *μ*g MeHg. Although this dose did not affect the overall morphology of the spinal cord, we demonstrated here that it is related to a reduction in thickness of the spinal cord layers, as well as to impairments in cell cycle proteins and in early neuronal differentiation.

In fact,* in ovo* development is a good model for toxicity studies because embryos develop in the absence of maternal factors, which may compromise the assay results. The metal injection in the yolk sac is effective for neurodevelopmental toxicology, and these assays have been validated in our previous studies with lead acetate and MeHg [23, 28, 31]. Moreover, the experimental design is also important, particularly the establishment of the exposure time. Preliminary tests (data not shown) using a shorter exposure times than the 7 days adopted here were not sufficient for the incorporation of MeHg by embryos. Additionally, considering the exposure time, the choice of treatment and analysis ages are equally essential.

In this study, we exposed embryos in earlier stage of development of CNS (E3), soon after the neural tube closure. At this stage, neural tube is composed by a nondifferentiated neuroepithelium. Then, to understand the neurodevelopmental toxicity of MeHg, cellular analyses were performed at E10, when the layers of the spinal cord are distinguished and composed by neuronal and glial differentiated cells. The used time gap between exposure and analyses was calculated in order to assess the period of vulnerability of embryos and then to assess the effects of MeHg on essential cell mechanisms, which are inherent to development of spinal cord.

Our results showed that MeHg causes a reduction in the thickness of the layers, reflecting the neurotoxicity of this metal in the spinal cord tissue. Carvalho et al. [23] demonstrated the deposition of MeHg in the layers of the cerebellum of chicken embryos and also showed morphological changes in the Purkinje layer in the first postnatal week. Studies about MeHg poisoning have shown the effects of MeHg on the cytoarchitecture of CNS layers in humans and animals, affecting the disposition and number of neurons, as well as on the size of the brain and cerebellum [6, 7, 32–35].

Regarding the effect on the morphology of CNS layers, we tested the hypothesis that MeHg causes impairments in cell proliferation, an essential mechanism of development. Then, we analyzed more specifically some proteins involved in cell cycle in order to better comprehend the cellular basis of MeHg toxicity. Our data showed a significant reduction in the number of proliferating neural cells in the ependymal and mantle layers. Neural cell proliferation was disturbed by MeHg, as demonstrated by* in vitro* and* in vivo* assays [14, 35, 36]. It was also demonstrated that MeHg affects the proliferation in all regions of the developing CNS, such as the spinal cord in* Xenopus laevis* and* Danio rerio* [37, 38], as well as in the murine brain and cerebellum [34, 39, 40].

The idea that MeHg compromises the cell proliferation was explored in classic works that focused on the mitosis inhibition related to the disruption of G1 and G2 progress [7, 34, 39]. Data about the expression of regulatory molecules involved in cell cycle checkpoints, such as p21 and cyclin, have been demonstrated mainly in the brain [14] and hippocampal [35] and cerebral cortex [40, 41]. p21 protein plays a central role in cell cycle arrest in response to DNA damage by inhibiting the initiation of replication in the S phase. As expected, our results showed an increase in the expression of p21 in the ependymal and mantle layers after MeHg exposure. Ou et al. [42] and Faustman et al. [43] also observed this behavior of p21 in neural cells after MeHg treatment. However, here we found the association of a decrease in cell proliferation with an increase of p21 expression in the developing spinal cord. Taken together, these data suggest the cellular impairment caused by MeHg and the fact that this organometal causes the arrest of the cell cycle in G1. Interestingly, regarding the interactive role of cyclin E and p21, the decrease of cyclin E was expected after the MeHg treatment, as demonstrated by Burke et al. [14], Falluel-Morel et al. [35], and Xu et al. [40]. In fact, cyclin E has been identified as a target of MeHg, reducing its expression in brain development. On the other hand, our data showed that MeHg did not change the cyclin E expression in the spinal cord of chicken embryos.

The upregulation of p21 is required in response to DNA damage. Indeed, using *γ*- H2A.X as a marker of DNA damage, we verified the occurrence of DNA double-strand breaks in the spinal cord of MeHg-treated embryos, demonstrating the genotoxic effect of this metal. The DNA damage may cause sequences of intracellular signaling that contribute to the upregulation of p21, and unrepaired damage may cause signaling to apoptosis. Here, we found that MeHg induces apoptosis, recognized by DNA fragmentation. Apoptotic cells were observed mainly in the mantle layer, the same layer where *γ*-H2A.X-positive cells were found. This combination of data reinforces the argument that the toxicity of MeHg seems to activate the signaling cascade of programmed cell death or apoptosis in both adult [44–46] and developing CNS [6, 14, 22, 35, 38]. Moreover, we proposed that the association between decreased proliferation and increased apoptosis may act as one cause of the reduction in the thickness of the spinal cord layers. Additionally, this impairment can progress during embryonic development, as well as in childhood and adulthood phases.

Considering the fact that the mantle layer appears be the more affected by MeHg and also that neurons are well recognized in this layer at the embryonic age evaluated, we investigated whether MeHg interferes in the expression of *β*-tubulin III, a marker of differentiated neurons. In spite of our expectation, the dose used did not compromise this protein. However, when we analyze the expression of NeuN, a neuron-specific nuclear protein, which is recognized in postmitotic neurons and/or during neuronal differentiation, we observed a significant decrease on the expression of this protein. Our results showed that MeHg affects differentially the neuron maturation, in the same embryonic stage. This can be explained, considering that to organize the spinal cord layers during development, the cells need to differentiate and migrate in different rhythms. Thus, in the same embryonic stage, we found both early and late phases of neurogenesis. In general, our results provide new insights in attempt to contribute to better understanding the cellular basis of complex MeHg neurotoxicity, in developing spinal cord.

## 5. Conclusion

The basis of how MeHg acts during the spinal cord development is incompletely described. From toxicological point of view, these results are very important because they showed for the first time that* in ovo* MeHg exposure alters spinal cord development by causing DNA double-strand breaks and also disturbing the mechanisms of proliferation and cell death, differentially interfering in early and late neurogenesis phases.
